# The effect of whole-brain radiation (WBI) and Karnofsky performance status (KPS) on survival of patients receiving stereotactic radiosurgery (SRS) for second brain metastatic event

**DOI:** 10.1007/s13566-016-0287-y

**Published:** 2016-12-06

**Authors:** D. R. Brown, R. Lanciano, C. Heal, A. Hanlon, J. Yang, J. Feng, M. Stanley, R. Buonocore, A. Okpaku, W. Ding, S. Arrigo, J. Lamond, L. Brady

**Affiliations:** 1 Philadelphia CyberKnife, Crozer-Keystone Health System, 2010 W Chester Pike #1050, Havertown, 19083 PA USA; 2 Drexel University College of Medicine, Philadelphia, PA USA; 3 University of Pennsylvania, Philadelphia, PA USA

**Keywords:** Brain metastasis, Stereotactic radiosurgery (SRS), Whole brain irradiation (WBI), CyberKnife

## Abstract

**Objective:**

The objective of the present study is to analyze prognostic factors affecting survival of patients receiving stereotactic radiosurgery (SRS) for second brain metastatic event (SBME) following initial treatment with whole brain irradiation (WBI), surgical resection, or previous SRS.

**Methods:**

The 88 patients treated with SRS for SBME at Philadelphia CyberKnife between January 2006 and October 2013 were included in the study group. Cox proportional-hazards regression was used to identify prognostic factors that significantly impacted survival from the time of SRS for SBME. Independent variables considered in survival analysis included primary disease, first brain metastatic event (FBME) treatment type, age, gender, number of brain metastases at SBME, Karnofsky performance status (KPS), recursive partitioning analysis (RPA), and presence of extracranial metastasis.

**Results:**

The median survival for all patients was 7.31 months. Log-rank comparison of Kaplan-Meier survival curves revealed significant impact by Karnofsky performance status (*p* = 0.003), RPA class (*p* = 0.008), age (*p* = 0.014), and FBME treatment type (*p* = 0.010). Median survival was longer for patients who had not previously received WBI (14.7 months). Median survival was further increased in patients who had not received previous WBI and demonstrated KPS scores of 70–100 (19.5 months). Patients who received WBI prior to SBME treatment experienced a pronounced decrement in median survival (5.7 months), yet patients in this group who demonstrated strong KPS scores (80–100) experienced significantly increased survival (15.5 months).

**Conclusions:**

The outcomes of SRS for SBME are most favorable for patients who have not received previous WBI or who have maintained higher performance status despite previous WBI.

## Introduction

The clinical management of brain metastases has long focused on local brain control for maintenance of neurologic function, since survival has been limited with whole brain radiation for the primary treatment of multiple metastases. [1] Improved survival and quality of life has been made possible through the development of improved diagnostic tools, surgical techniques, and highly precise stereotactic radiosurgery (SRS) options. [1] Choosing high-tech and costly treatment should be reserved for those most likely to benefit from that treatment. Therefore, survival prediction tools using clinical prognostic factors best guide treatment recommendations.

New cancer diagnoses exceed 1.6 million per year as of 2016 [2]. The rate of brain metastases from all cancers is approximately 100,000–170,000 per year or nearly 10% of all patients and as high as 25% in lung cancer patients [3]. Patients are living longer due to improvements in systemic therapy, leading to a higher prevalence of brain metastasis, which remains a sanctuary site for most drugs [3]. The treatment of brain metastases has historically been limited to surgical resection and/or whole-brain radiotherapy (WBRT), with survival of non-surgical candidates approximately 3–4 months [4]. With the development of more precise treatment modalities like SRS, the inherent long-term risk of neuro-cognitive decline from WBRT can be avoided [1]. In addition, brain control and possibly survival is enhanced with limited brain metastatic disease after surgery or whole brain radiation with SRS as adjuvant or boost [5].

While there exist multiple diagnosis-specific survival prediction tools for patients presenting with first brain metastatic event (FBME), survival data for patients experiencing a second brain metastatic event (SBME) is lacking. The Radiation Therapy Oncology Group (RTOG), using recursive partitioning analysis (RPA), formed a regression tree based on significant prognostic factors from 18 pretreatment and 3 treatment factors from completed trials of patients with brain metastases treated with whole brain radiation [6]. Following the verification of the RPA prognostic index, further refinement based on treatment was developed for radiosurgery with the Score Index for Radiosurgery (SIR). The SIR was developed based on classical parameters including age, Karnofsky performance status, systemic disease control, number of intracranial lesions, and size of lesions [7]. Lorenzoni et al. simplified this system using only the Karnofsky performance status, presence of extracranial metastases, and primary tumor control as prognostic factors to estimate prognosis [8]. Lorenzoni’s prognostic assessment index, the Basic Score for Brain Metastases (BSBM), was shown to be equivalent to the SIR but in simplified form.

The most useful prognostic assessment index, the Graded Prognostic Assessment (GPA) index, reported by Sperduto et al., compared the existing prognostic indices including their new GPA index using data from 1960 treated patients in the RTOG database. The GPA index, similar to previous indices, includes age, Karnofsky performance status, and extracranial metastases as well as number of brain metastases [9]. Sperduto et al. updated their GPA index to a Diagnosis-Specific Graded Prognostic Assessment (DS-GPA), with diagnosis-specific prognostic factors estimating survival times [10]. The DS-GPA provides the most accurate estimates of survival from the time of FBME treatment but was not designed to provide estimates for patients experiencing SBME.

The purpose of the present study is to analyze prognostic factors affecting survival of patients receiving SRS for SBME following initial treatment with WBI, surgical resection, or previous SRS for FBME.

## Methods

### Patient review

All patients treated for brain metastasis with SRS at Philadelphia CyberKnife between January 2006 and October 2013 were reviewed (229 patients). Only patients treated with SRS for SBME were included in the study group. The study group consisted of 88 patients who received one of the following: SRS for SBME ≥2 months after WBI, surgical resection, or SRS for FBME and for whom follow-up/survival information was available. Only three patients were lost to follow-up.

### Data collection

Further data was collected on the 88 patients that received SRS for SBME on this IRB-approved study. Selection of prognostic factors of interest was guided by established prognostic indices for initial brain metastasis treatment [6–10]. Pretreatment and treatment factors evaluated include age, primary cancer type, presence of extracranial metastasis, FBME treatment type, Karnofsky performance status (KPS), recursive partitioning analysis (RPA), number of intracranial lesions at the time of SBME, total SRS clinical treatment volume (CTV), dose and number of fractions, and treatment margins. Dose prescription was guided by size of the brain metastasis as per the Radiation Therapy Oncology Group (RTOG) studies. Follow-up and survival data was collected through various sources including Philadelphia CyberKnife charts, referring physician charts, Crozer-Keystone Health System electronic medical records, and obituaries. Patients were followed every 3 months by the patient’s neurosurgeon, radiation oncologist, or medical oncologist. Date of death or last follow-up was recorded for all patients.

### Statistical analysis

Descriptive statistics were used to characterize the study group in terms of demographics and treatment characteristics, including mean, SD, median, and range for continuous variables, as well as frequencies and percentages for categorical variables. Univariate cox proportional-hazards modeling was used to identify prognostic factors significantly impacting survival from the time of SRS treatment for SBME. Hazard ratios were estimated, along with 95% confidence intervals. Multivariable regression modeling was accomplished using cox regression and considering variables significant in the univariate analysis at the 0.20 level, followed by manually removing factors one at a time on the basis of least significance, until only those remaining demonstrate significance at the 0.10 level. Kaplan-Meier survival estimates were then generated for all patients and for prognostic subgroups identified in the cox regression analysis. Comparisons in overall survival by group were accomplished using log-rank statistics. Finally, median survival and interquartile range estimates based on Kaplan-Meier methodology are presented for important subsets of patients identified by multiple predictors.

## Results

### Demographics

The median and mean ages of the patient population were 59.3 and 59.6 years at the time of SBME treatment with 65% female and 35% male patients. More than 50% of the study group had primary lung cancer with multiple other cancers observed. Over 70% of patients had WBI as part of FMBE (Table 1). Median time from WBI to SRS for SBME was 8.5 months (2.2–38.4 months). The median planning target volume (PTV) dose was 20 Gy (13.5–30 Gy) delivered in one fraction (one to five fractions) with a median 1.25-mm margin around the clinical target volume (CTV). Additional treatment characteristics are presented in Table 2.

**Table 1:** Patient demographics

Variable	Category	Frequency	Percent
Age	Median 59.3 years (range 32.8–86.2 years)		
	Mean 59.6 years (SD ±11.44 years)		
Sex	Male	31	35.2
	Female	57	64.8
Primary cancer	Lung	50	56.8
	Breast	16	18.2
	Colorectal	8	9.1
	Melanoma	6	6.8
	Renal cell	3	3.4
	Gynecologic	3	3.4
	Mesothelioma	1	1.1
	Sarcoma	1	1.1
KPS score	90–100	13	14.8
	70–80	42	47.7
	<70	33	37.5
No. of brain metastasis	1	31	35.2
	2–3	39	44.3
	>3	18	20.5
Extracranial metastasis	Present	39	44.3
	Not present	49	55.7
RPA class	1	25	28.4
	2	30	34.1
	3	33	37.5
FBME treatment	WBI	62	70.5
	Surgery	18	20.5
	SRS	8	9.0

**Table 2:** Treatment characteristics

Variable	Median (range)
CTV volume (cc)	2.64 (0.09–52.44)
PTV volume (cc)	3.69 (0.16–52.44)
Margin (mm)	1.25 (0–3.0)
PTV dose (Gy)	20 (13.5–30)
Fractions	1 (1–5)
No. of beams	134 (53–415)
Isodose (%)	70 (54–82)
BED (Gy)	50.4 (19.6–65.1)

### Overall survival

**Fig. 1:**
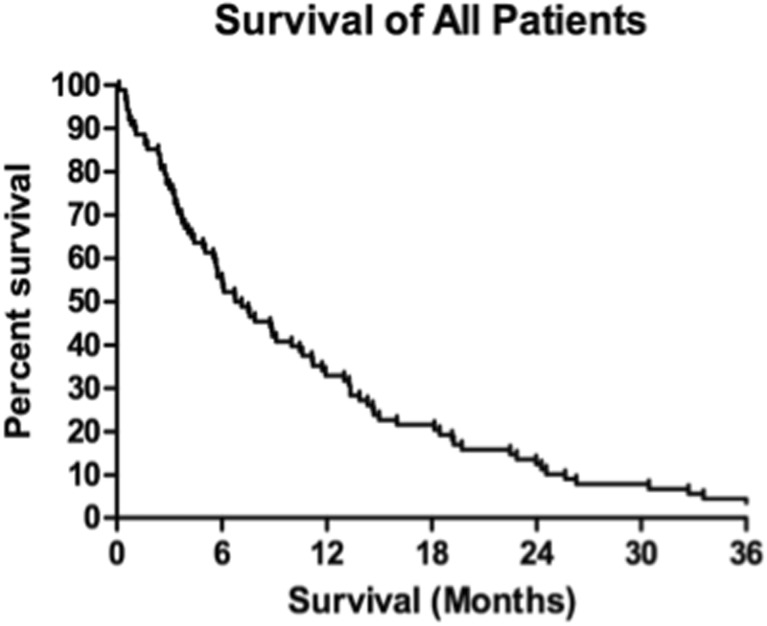
Kaplan-Meier overall survival estimates for all patients

**Fig. 2:**
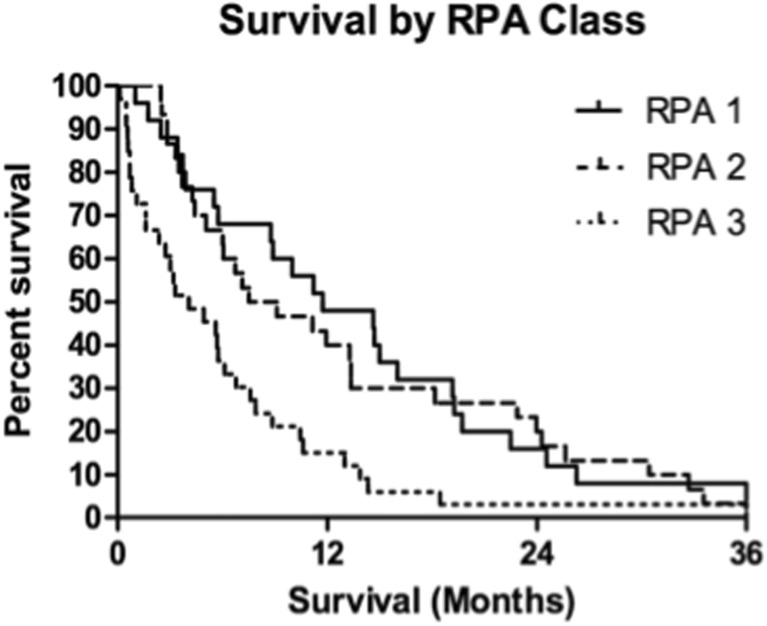
Kaplan-Meier overall survival estimates for all patients by RPA class (*p* = 0.008)

**Fig. 3:**
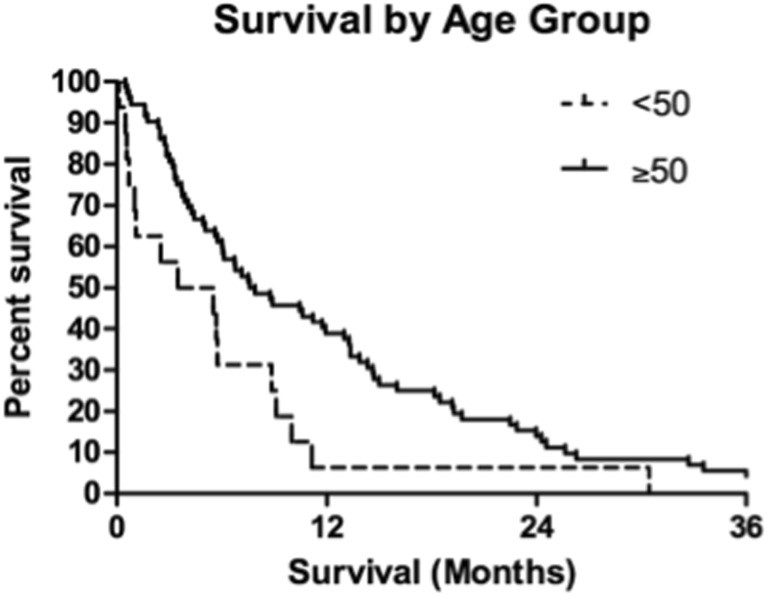
Kaplan-Meier overall survival estimates for all patients by age group (*p* = 0.01)

**Fig. 4:**
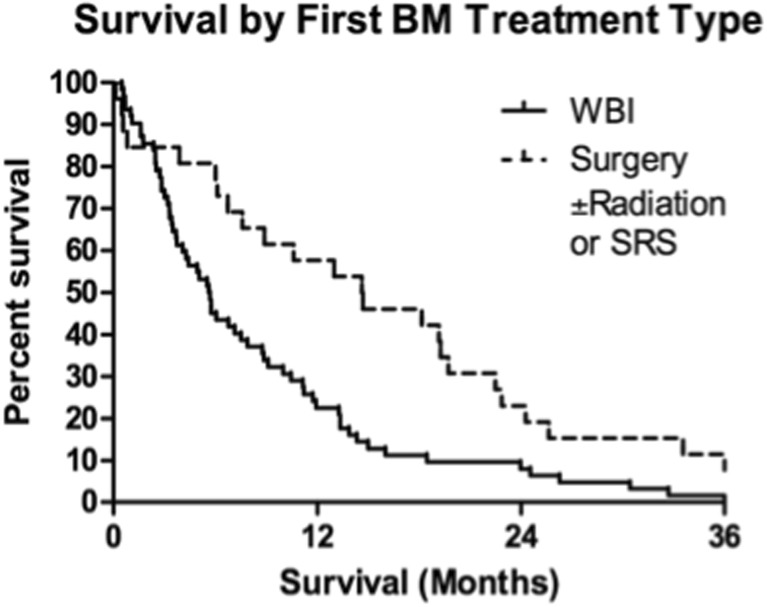
Kaplan-Meier overall survival estimates for all patients by first BM treatment type (*p* = 0.01)

**Fig. 5:**
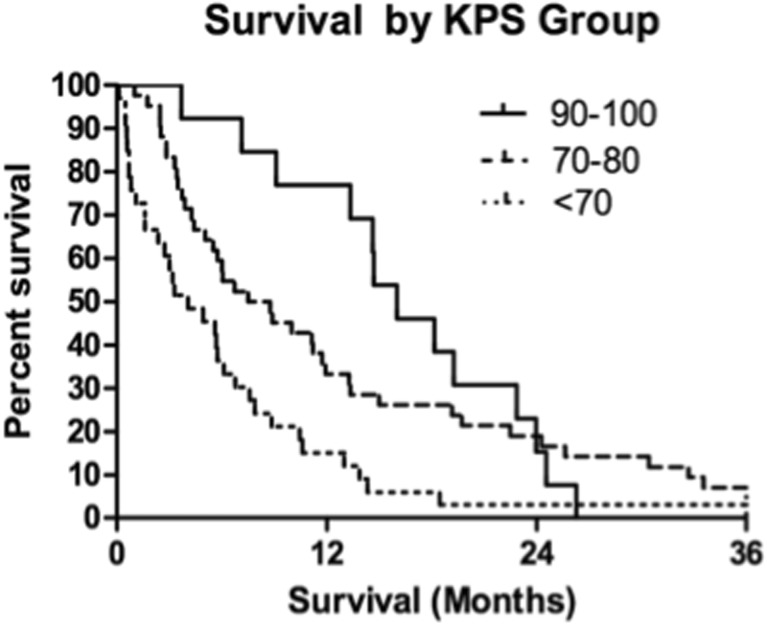
Kaplan-Meier overall survival estimates for all patients by KPS (*p* = 0.003)

Kaplan-Meier estimates showed 1- and 2-year actuarial survival of all patients of 36 and 16%, respectively (Fig. 1). At the last follow-up, there were 14 patients living of the 88 total patients. There were four patients alive after the 3-year follow-up and one patient alive after the 6-year follow-up. Log-rank tests identified prognostic factors with significant impact on survival. Of the pretreatment factors analyzed, RPA class, age, previous WBI, and KPS score impacted survival, with *p* values of 0.008, 0.014, 0.010, and 0.003, respectively (Figs. 2, 3, 4, and 5). Number of brain metastasis at time of SBME, presence of extra-cranial metastasis, and primary diagnosis were not significant prognosticators (Table 3). Multivariate analysis revealed that only KPS (*p* = 0.001) and age (*p* = 0.011) were significant when all significant factors were added to the model.

**Table 3:** Results from univariate cox proportional-hazards modeling of prognostic factors for survival

Prognostic factor		Hazard ratio	95% LCL	95% UCL	*P* value
Age	<50	2.096	1.164	3.777	0.014
	>50^a^	1.000	–	–	–
ECM	No	0.688	0.436	1.084	0.107
	Yes^a^	1.000	–	–	–
WBI	No	0.456	0.246	0.847	0.010
	Yes^a^	1.000	–	–	–
KPS score	<70	0.336	0.163	0.694	0.003
	70–80	0.466	0.283	0.767	0.003
	90–100^a^	1.000	–	–	–
No. of mets	1	1.139	0.604	2.150	0.730
	2–3	1.344	0.724	2.496	0.399
	>3^a^	1.000	–	–	–
Primary diagnosis	Breast	1.443	0.601	3.462	0.412
	Colorectal	0.957	0.345	2.656	0.933
	Lung	1.260	0.61	2.602	0.532
	Melanoma	2.196	0.776	6.214	0.138
	Other^a^	1.000	–	–	–
RPA	1	0.367	0.203	0.665	0.001
	2	0.484	0.284	0.824	0.008
	3^a^	1.000	–	–	–

^a^Reference category

Recipients of previous WBI had 1- and 2-year actuarial survival of 22.6 and 8.1% while patients who did not receive WBI experienced 1- and 2-year actuarial survival of 57.7 and 23%. KPS scores were stratified into three groups defined by previous literature review: <70, 70–80, and 90–100 [9, 10]. Lowest KPS score (<70) resulted in markedly decreased 1- and 2-year actuarial survival compared with higher scores (Table 4).

**Table 4:** One- and two-year actuarial survival of significant prognosticator groups

Prognostic factor		One-year	Two-year
WBI	No	57.7%	23.0%
	Yes	22.6%	8.1%
KPS score	90–100	76.9%	15.4%
	70–80	33.3%	19.0%
	<70	15.1%	3.0%
RPA class	1	48.0%	16.0%
	2	40.0%	20.0%
	3	12.1%	3.03%
Age	<50	6.25%	6.25%
	>50	38.9%	13.9%

**Table 5:** KPS comparison: current study vs Sperduto et al. RTOG analysis [10]

	Current Study	RTOG Analysis
<70	37.5%	14.9%
70–80	47.7%	43.5%
90–100	14.8%	41.6%

### Median survival

The overall median survival of the 88 patients receiving SRS for SBME was 7.3 months (IQR 3.3–14.6 months). Low KPS score (<70) at the time of SBME treatment had a significant detrimental impact on survival, lowering median survival to 4.08 months (IQR 1.1–7.9 months) compared to 8.83 months (IQR 4.0–18.1 months) for patients with KPS 70–80 and 16.01 months (IQR 13.3–22.9 months) for patients with KPS 90–100. Patient age proved to be a significant prognosticator when patients were grouped above and below 50 years. Younger patients demonstrated significant survival decrement with a median survival of 4.5 months (IQR 0.9–8.9 months) compared with 7.7 months (IQR 3.7–16.5 months) for patients 50 and older. RPA class significantly predicted survival with median survivals of 11.7, 8.3, and 4.1 months for classes 1, 2, and 3, respectively.

### Subgroup analysis by previous WBI and KPS score

**Fig. 6:**
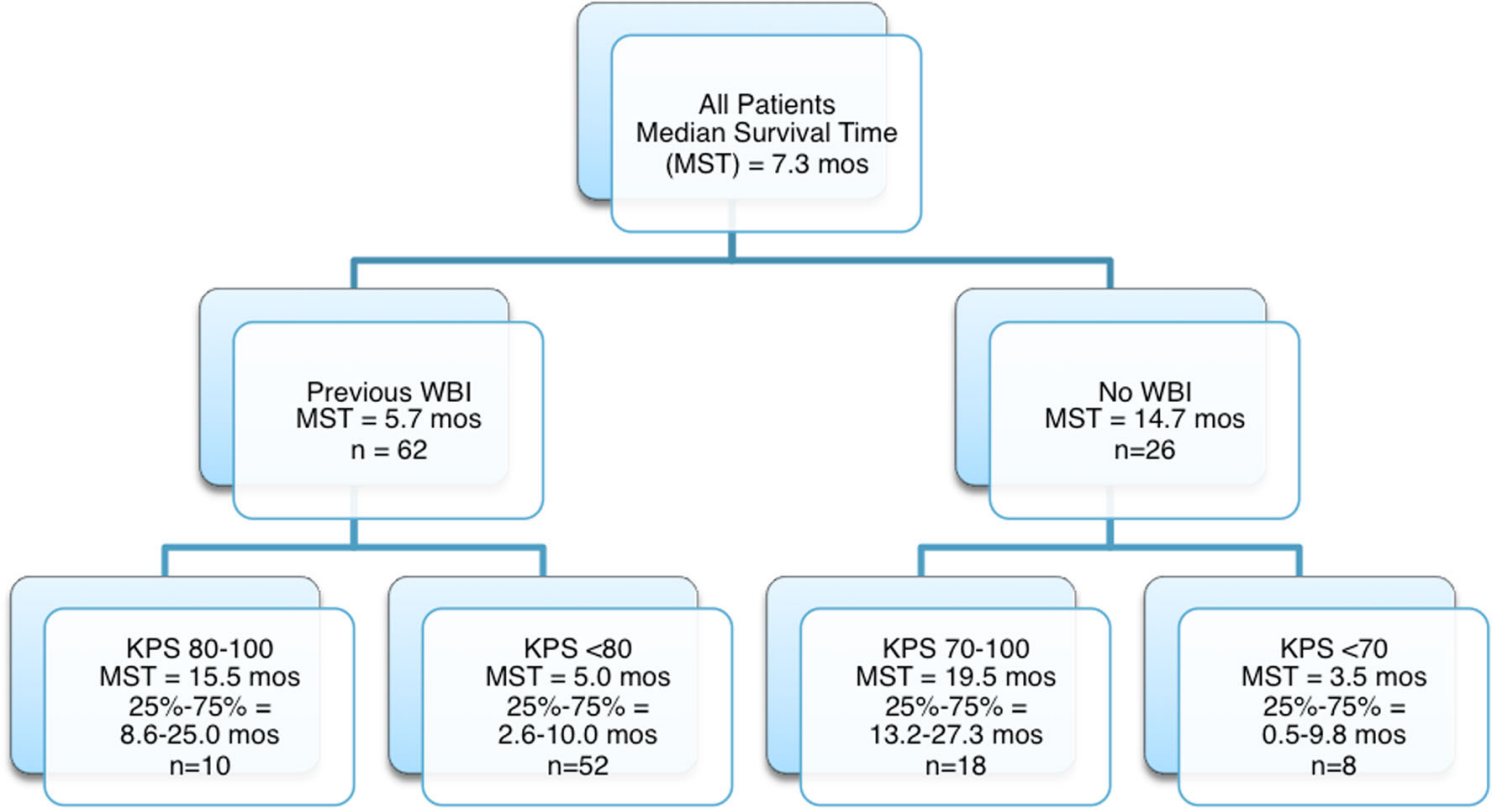
Subgroup analysis defined by previous WBI and KPS score

**Fig. 7:**
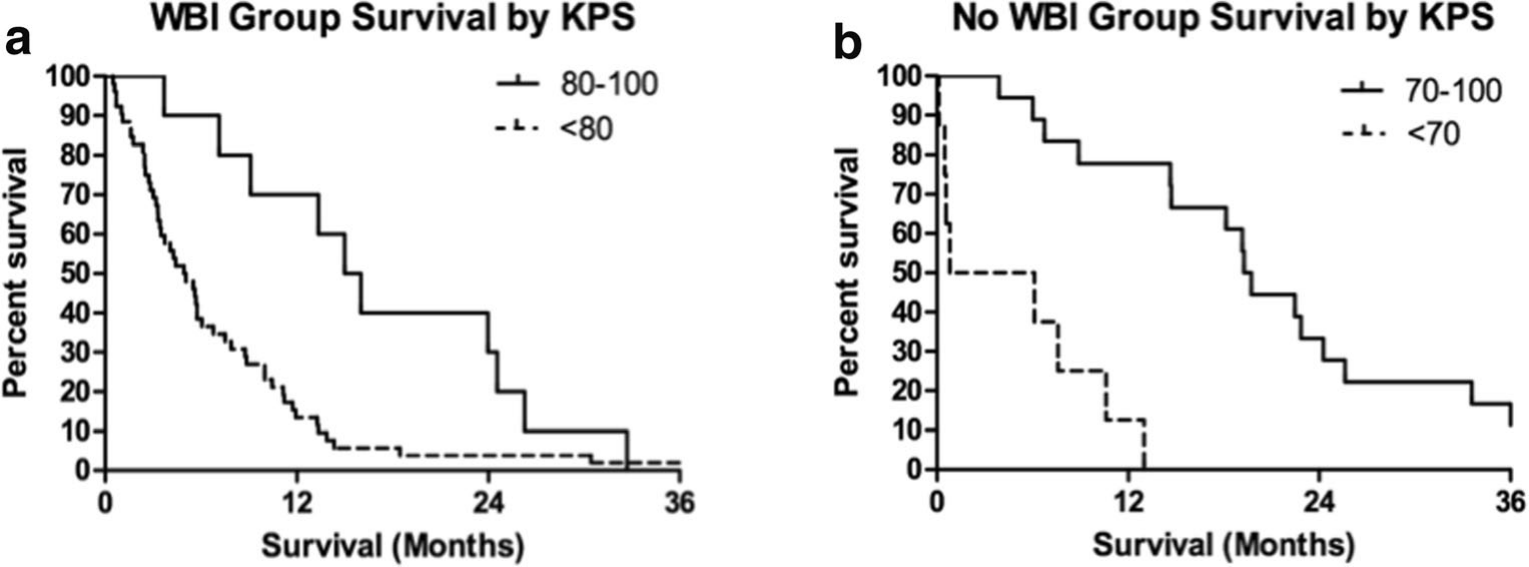
**a** Kaplan-Meier overall survival estimates for WBI patients by KPS score (*P* = 0.005). **b** Kaplan-Meier overall survival estimates for no. of WBI patients by KPS score (*P* = 0.0001)

Patient age, despite being a significant factor in multivariate analysis, did not provide significantly distinct survival groups when used as either the primary or secondary stratification criteria because of the small number of patients less than 50 years old. Primary stratification by previous WBI and secondary stratification by KPS score provided four groups of patients with significantly distinct median survival estimates. WBI had the most significant negative impact on survival, decreasing median survival from 14.7 months (IQR 6.3–22.8 months) for patients who did not receive WBI to 5.7 months (IQR 3.1–11.6 months) for those who did receive WBI (Fig. 6). Secondary stratification by KPS score within these two groups showed the cumulative effect of both significant prognosticators on survival. For recipients of WBI, the greatest impact of KPS was seen between patients scoring <80 and those scoring 80–100, *p* value 0.005 (Fig. 7). KPS scores below 80 decreased median survival to 5.0 months compared with 15.5 months for patients scoring 80–100 (Fig. 6). For patients who did not receive previous WBI, KPS score had a similar effect on median survival with the greatest impact seen between patients scoring <70 and those scoring 70–100, *p* value <0.001 (Fig. 7b). Scores below 70 decreased median survival to 3.5 months while scores 70–100 resulted in the longest median survival of any group at 19.5 months (Fig. 6).

## Discussion

The observed 7.3-month overall median survival for patients receiving SRS for SBME in the present study is nearly identical to the 7.23-month overall median survival observed in the multi-institution RTOG analysis that led to the creation of the DS-GPA [10]. Sperduto et al. reported on 4259 patients treated for initial brain metastasis with various combinations of surgery, WBI, and SRS [10]. The percentage of patients in the present study who received previous WBI is 70%, similar to the 76% of patients in the RTOG analysis who received WBI. The current study group demonstrated poorer average pretreatment performance status than the RTOG group with 14.8% KPS 90–100 compared with 41.6% in the RTOG group and 37.5% KPS <70 compared with 14.9% in the RTOG group (Table 5). Interestingly, patients in the current study who received SRS for SBME, with treatment history similar to the Sperduto et al. study group treated for FBME, experienced nearly identical overall median survival despite a lower average performance status.

Our results are consistent with previous reports of SRS for SBME. Our overall median survival is comparable to Kurtz et al. who reported a median survival of 11.7 months after SRS for SBME in a group of healthier patients (87% ECOG 0–1/KPS 70–100), most of whom received WBI for FBME (81.1%) [11]. Kurtz et al. reported RPA class as a significant prognosticator for survival following SBME [11]. We also found RPA to be significant for predicting survival from SBME in univariate analysis, but not in multivariate analysis. KPS was the most significant factor in our database in multivariate analysis and represents a powerful and simple prognosticator for SBME. KPS has consistently proven to be a significant prognosticator in all indices for both first and second brain metastatic events [6–10].

Our series compares favorably (median survival of 14.7 months) for patients who did not receive previous WBI treated with second-course SRS for SBME to Minniti et al. who reported median survival of 10.3 months [12]. Additionally, our series is comparable (median survival of 5.7 months) for patients who did receive previous WBI treated with SRS for SBME to Harris et al. who reported median survival of 5.9 months [13]. WBI for FBME has not improved survival in randomized trials [14–16] over surgery or SRS alone. A recent randomized trial for patients with one to three brain metastases suggest that WBI provides no survival benefit over SRS alone for treatment of FBME. Greater cognitive decline was associated with WBI than with SRS alone [17]. Our study suggests that previous WBI also decreases median survival for patients treated with SRS for SBME. It is not clear if WBI directly affects survival due to decrease in KPS secondary to decline in cognitive function or is an affect of selection for more advanced disease requiring WBI for FBME and cannot be addressed in the current study.

## Conclusion

KPS is the most significant prognosticator of survival from SBME when treated with SRS. Use of WBI for FBME treatment was associated with significant survival decrement from the time of SBME treatment with SRS. Median survival in our series of patients treated with SRS for SBME is comparable to that of FBME series. However, our results do not account for the inherent selection bias of retrospective studies and future prospective trials should verify these observations.
